# Comparative effects of dietary muramidase and phytogenics on the growth performance and gastrointestinal functionality of broiler chickens

**DOI:** 10.1016/j.psj.2024.104147

**Published:** 2024-07-31

**Authors:** Necmettin Ceylan, Cristiano Bortoluzzi, Oguz Gunturkun, Estefania Perez-Calvo

**Affiliations:** ⁎ Department of Animal Science, Faculty of Agriculture, Ankara University, Ankara, Türkiye; † dsm-firmenich, Animal Nutrition and Health, Kaiseraugst, Switzerland; ‡ dsm-firmenich, Animal Nutrition and Health, İstanbul, Türkiye

**Keywords:** muramidase, phytogenics, broiler, gastrointestinal functionality, peptidoglycan

## Abstract

The objective of the present study was to compare the effectiveness of dietary supplementation of muramidase (**MUR**) and 2 phytogenic additives on the growth performance, intestinal morphology, bacteria load, and production of short-chain fatty acids (**SCFA**) of broiler chickens raised under field-like conditions. A total of 6,400 day-old Ross 308 broiler chicks were randomly selected and distributed into 32 floor pens, with 200 chicks (100 males and 100 females)/pen. The treatment groups were an unsupplemented control, and the experimental groups supplemented with MUR at 35,000 LSU(F)/kg of feed, phytogenic 1 (Phyto 1, based on thymol) at 100g/ton feed, or phytogenic 2 (Phyto 2, based on alkaloids) at 60g/ton feed, for a total period of 41 d. A 4-phase feeding program was applied (starter, grower, finisher and withdrawal). The paramenters evaluated were: growth performance, carcass yield, concentration of muranic acid in the jejunum content and excreta, liver enzyme concentration, intestinal morphology, and bacteria enumeration and short and branch chain fatty acids (SCFA and BCFA) in the cecal content. Data were analyzed by ANOVA and Tukey's test was used to separate the means. Soluble muramic acid (MurN) in the jejunum increased with the supplementation of MUR and Phyto 2 when compared to the other groups (*P* = 0.0001), but only the supplementation of MUR increased the concentration of MurN in the excreta. The supplementation of all feed additives improved the body weight gain and the body weight corrected feed conversion ratio when compared to the control group (*P* = 0.0001). MUR increased villus heigh (**VH**) when compared to the control or the other supplemented groups (*P* = 0.0001), and led to the highest concentration of most SCFA, total BCFA, and total SCFA (*P* < 0.05). In conclusion, the supplementation of MUR and phytogenics to the diets of broiler chickens improved the growth performance, but MUR, only, was capable of effectively degrading peptidoglycans (**PGNs**) in both intestinal segments, as well as to increase the abundance of beneficial bacteria and SCFA production.

## INTRODUCTION

The digestive system in broiler chickens has a very complex ecosystem as it hosts many beneficial and harmful micro-organisms as well as digestion and absorption. *Escherichia coli, Clostridium, Salmonella, Eimeria* and *Campylobacter* spp. are the dominant pathogens in the digestive system and pose serious risks for intestinal health, and if not controlled, they adversely affect growth performance, development, feed efficiency and health, and may lead to the development of enteric diseases. The basic criterion of economical and sustainable chicken production is an optimal gastrointestinal functionality (Celi et al., 2017), and it is possible to suppress pathogenic microorganisms with adequate feed and feeding strategies.

Antibiotic growth promoters (**AGP**) have been used in the past due to their effects in preventing pathogens and their negative effects, but its increased restrictions, due to antibiotic resistance and related problems, has spread all over the world after it started with the European Union in 2006 (Castanon, 2007). In recent years, various products consisting on probiotics, prebiotics, phytogenics, organic acids and their combinations have been developed in order to improve the growth performance, intestinal integrity, nutrient digestibility, and prevent the negative effects of pathogenic microorganisms in broiler chickens. Another strategy that has been used in the last years is related to enzymes used in the feed that do not act on feed components, but on endogenous molecules present in the intestine of the animals, such as muramidase (MUR) that act on peptideoglycans (**PGNs**) from gram-positive bacteria. İt has been shown that an specific MUR is beneficial in improving the growth performance of broiler chickes to the same extent as AGP (Goes et al., 2022; Bortoluzzi et al., 2023). The mechanism of action of this enzyme has been fairly defined in several studies (Wang et al., 2021; Brugaletta et al., 2022; Goes et al., 2022; Amer et al., 2023; Bortoluzzi et al., 2023). By the degradation of PGNs in the intestine of the animals, which leads to a anti-inflammatory response, the dietary supplementation of this enzyme reduces the infiltration of inflammatory cells in the intestine and liver of coccidiosis induced broiler chickens (Bortoluzzi et al., 2023).

On the other hand, phytogenic feed additives constitue a mixture of volatile substances, in some cases, lipophilic (Hashemi and Davoodi, 2011), including hydrocarbons, ketones, phenols, and alkaloids. Many factors may influence their chemical composition, such as weather, season of harvest, storage, and others (Applegate et al., 2010). Most of the substance extracted from plants are secondary metobolites with an especific function to the metabolism of the plant (Hashemi and Davoodi, 2011). The mechamins of action of phytogenic feed additives varies depending, for example, on the position of the hydroxyl group. Thymol and carvacrol are isomeric molecules with similar antimicrobial effects (Diaz-Sanchez et al., 2015), with immunomodulatory effects on the host, that maintain a balance between cellular and humoral immune response (Hashemi and Davoodi, 2012). Additionally, alkaloids have also been investigated for their beneficial effects in broilers, especially by their anti-inflammatory and antimicrobial effects, and potential to replace AGPs (Ceylan et al., 2023).

Since the mechanism of action of different feed additives may vary depending on various factors, the hypothesis of this study was that the supplementation of MUR and phytogenics would improve the growth performance by improving the gastrointestinal functionality parameters of broiler chickens. Therefore, the objective of the present study was to compare the effectiveness of dietary supplementation of MUR and 2 different phytogenic additives on the growth performance, intestinal morphology, bacteria load, and production of short-chain fatty acids (**SCFA**) of broiler chickens raised under field-like conditions.

## MATERIALS AND METHODS

The authors confirm that the ethical policies of the journal, as noted on the journal's author guidelines, have been adhered to and the appropriate ethical review committee approval has been received. The US National Research Council's guidelines for the Care and Use of Laboratory Animals were followed. The Animal Ethics Committee of the Ankara University (Approval number: 2021-94) approved all procedures involving animals.

### Animal Breeding and Management

The research was carried out for 41 d in Beypiliç Broiler R&D farm, Turkey. A total of 6,400 day-old Ross 308 broiler chicks were randomly selected and their live weights were determined, and were randomly distributed into 32 floor pens (6.5*2 m each), with 200 chicks (100 males and 100 females)/pen. The broiler research house is a large-scale R&D facility of one of the largest broiler integrations in Turkey. The R&D facility has the capacity for 12,000 broiler (60 pens with capacity of 200 birds/pen) chickens and has similar environment and disease challenges as other houses in the integration. The house is equipped with a computer-controlled automatic tunnel ventilation system (Fancom, Lumina Farm Manager, Model 38), nipple drinkers, and computer-controlled suspended feeders, and wood shavings were used as new litter on the floor. In the study, crumble feed was used from 0 to 10 d, and thereafter pellet feed, during grower, finisher and withdrawal phases, and water were provited *ad libitum*. The house temperature was set at 33°C at the placement, and gradually decreased to 23°C until day 21st and kept at this level with automatic ventilation and heating systems until the end of the experiment following Ross recommendations. The lighting with 20-30 lux fluorescent lamps, 23 h of light and 1 h of darkness in the first week, was then reduced to 5 to 10 lux and continued as 20 hours of light and 4 h of darkness.

The broiler chickens used in the current experiment were vaccinated against Infectious Bronchitis (**IB**), Newcastle Disease (**ND**), and gumboro disease in the hacthery, and against IB and ND on d 11; ND vaccine was also applied via water on d 20.

### Trial Design, Feed, and Diets

The research was carried out with 4 treatment groups each with 8 replicates of 200 broiler chickens/replicate, which were the unsupplemented control, and the experimental groups supplemented with MUR at 35,000 LSU(F)/kg of feed (Balancius, dsm-firmenich, Switzerland), phytogenic 1 based on thymol (Phyto 1), and phytogenic 2 based on alkaloids (Phyto 2) in accordance to the manufacturer's recommendations. The feed additives used in the research and it's recommended feed application level, based on the manufacturer recommendations, in each phase are given in Table 1. Sunflower meal, wheat and poultry oil were included at low levels in the feeds based mainly on corn soybean meal and full-fat soybean. In the experimental diets, no growth-promoting additives were included. Xylanase and super-dose of phytase were included with the respective nutrient matrix. A 4-phase feeding program was applied (starter, grower, and finisher and withdrawal). The composition and nutrient content of the basal diets used in the periods are given in Table 2.

**Table 1:** Feed additives used in the experiment and its inclusion levels in tons of feed, kg/ton.

Treatments	Application dose, kg/ton		
	Starter	Grower	Finisher/Withdrawal
Control	0.00	0.00	0.00
Muramidase^1^	0.35	0.35	0.35
Phytogenic 1^2^	0.10	0.10	0.10
Phytogenic 2^3^	1.00	1.00	1.00

1 Muramidase:The commercial product named Balancius® Muramidase (EC 3.2.1.17) is an enzyme produced by a *Trichoderma reesei* strain, expressing a muramidase gene isolated from *Acremonium alcalophilum*. Each g contains 60,000 LSU(F) of activity. The research was carried out by adding the product in powder form at a level of 350 g to a ton of mixed feed.

2 Phytogenic 1: This products consists mainly of essential oil Thymol in granulate powder form, off-white colour. The minimum amount of thymol declared is 63,000 mg/Kg, while the analysed content is 88,200 mg/kg used in the experiment at the dosage rate of 100 g per ton of mixed feed.

3 Phytogenic 2*:* This product contains alkaloids from *Macleaya cordata*, and recommended to be used at 60 g/ton of feed. The declared contents of this commercial product at 1.0 kg/ton are: 600 mg *Macleaya cordata excracts* (quaternary benzo[*c*]phenanthridine alkaloids ) *and 36,000 mg* intact aerial parts diluted with wheat bran and calcium carbonate and lignosulpate to obtain 1 kg premix for uniform mix in the final broiler feed.

**Table 2:** Ingredient and nutrient composition of basal (control) diets (g/kg, as-fed basis)*.

Ingredients	Starter	Grower	Finisher	Withdrawal
Corn	490.0	514.4	601.9	585.0
Soybean Meal, 46	281.1	227.7	168.2	125.0
Full Fat Soybean, 35	100.0	90.0	90.0	90.0
Sunflower Meal, 36	45.6	31.6	22.2	48.7
Wheat	38.6	75.0	50.0	96.1
Poultry Meal	0.0	24.0	27.0	23.0
Poultry Fat	12.0	13.0	16.5	13.0
Limestone	8.02	6.64	8.11	4.34
Dicalcium phosphate	7.55	2.59	0.50	0.00
DL-Methionine	3.35	2.82	2.64	2.17
Lysine HCL	2.67	2.14	2.26	2.47
Salt	2.50	2.50	2.45	2.44
Phytase-Xylanase Enzyme^2^	2.00	2.00	2.00	2.00
Mineral Premix^3^	2.00	2.00	2.00	2.00
Sodium Bicarbonate	1.32	1.00	1.50	1.70
L-Threonine	1.26	0.80	0.71	0.64
Vitamin Premix^4^	1.00	1.00	1.00	1.00
Monimax^5^	0.50	0.50	0.50	0.00
Choline Chloride, 75%	0.48	0.38	0.43	0.44
Total	1,000	1,000	1,000	1,000
Nutrients^1^				
Crude Protein,%	23.64(23.49)	22.45(22.06)	19.92(19.97)	19.09(18.75)
ME, kcal/kg	2,990	3,090	3,190	3,190
Crude Fat,%	5.02	5.21	5.73	5.314
Crude Fiber,%	4.08(3.61)	3.62(3.42)	3.29(3.09)	3.77(3.40)
Crude Ash,%	5.56(5.66)	4.88(5.09)	4.50(4.80)	3.96(4.01)
Dig. Methionine,%	0.64	0.57	0.53	0.48
Dig. Lysine,%	1.28	1.15	1.02	0.96
Dig. Met+Cys,%	0.95	0.87	0.80	0.75
Ca,%	0.90	0.81	0.81	0.64
P available,%	0.45	0.41	0.36	0.35
Dig. Arginine,%	1.38	1.27	1.09	1.03
Dig. Threonine,%	0.86	0.77	0.68	0.64
Dig. Isoleucine,%	0.87	0.81	0.70	0.66
Dig. Valine,%	0.95	0.90	0.80	0.76
Dig. Tryptophane,%	0.25	0.23	0.19	0.19
Electrolyte Balance Poultry	261.5	234.9	211.5	201.9

⁎ Treatment diets was obtained by the supplementation of the feed additives on the expense of Corn in all basal diets

1 Parenthesis indicates results of chemical analysis.

2 Each 2 kg of the enzyme premix was supplemented to provide 2,000 FTU OptiPhos Plus as Phytase, and 1,500 EPU Hostazyme X as xylanase, both from Huvepharma Inc., per kg of feed.

3-4 Supplied the following per kg of feed: 4.13 mg retinyl acetate; 125 g cholecalciferol; 100 mg dl-a-tocopheryl acetate; 3.5 mg menadione; 3.5 mg thiamine; 9 mg riboflavin; 20 mg calcium d-pantothenate; 65 mg niacin; 0.02 mg vitamin B_12_; 2.2 mg folic acid; 4.5 mg pyridoxine; 0.22 mg biotin; 120 mg manganese (Mn oxide);1.5mg iodine (Ca iodate); 25 mg iron (Fe sulphate); 16 mg copper (Cu sulphate); 110 mg zinc (Zn oxide); 0.3 mg selenium (Na selenite).

5 Microgranulated product combing monensin and nicarbazin.

Before the experiment started, raw materials were separated in separated silos or storage area in sufficient quantities. Experimental feeds were produced in Beypiliç Bolu Feed Mill and shipped to the poultry house by silobuses. The starter feed was weighed in separated sacks for each floor pens and distributed to the hanging feeders, while grower and finisher feeds were distributed as 30 kg batches into each pens by automatic feeding system with 50 g sensitivity. In the production of the treatment feeds, the additives were prepared as a pre-premix with using soy, dicalcium phosphate (**DCP**) and enzyme in the diet, then the premix was slowly added directly to the top by opening the mixer together with the other additives such as salt, sodium bicarbonate, DCP and marble powder. Amino acids and Vitamin-Mineral premixes were dosed with the microdosage unit. The feeds were processed in the conditioner at 1.6 to 2 bar pressure and 78 to 80°C temperature for an average of 120 s, then pelleted in the pellet press, and sent to the cooler for their production.

### Measurements

#### Growth Performance

In the study, live weight (**BW**) and body weight gain (**BWG**) between measurement periods were determined by weighing all chicks of each floor pen at the beginning of the trial, on the 11th d, 23rd d, and 41st d. At the beginning and end of each feeding period, the feed was weighed on the pen basis to determine feed intake (**FI**). Mortality was recorded daily with the weight of the dead birds. Feed conversion ratio (**FCR**) was determined by taking into account death weights, feed consumption and live weight gain for each period.

At the end of the experiment (d 41), all the birds were weighed after 6 to 8 h fasting period. All the birds were sent processed at the Beypiliç Broiler Slaughterhouse, where the sampled birds were properly identified. The birds were hanged, desensitized, and bled. After bleeding, the birds were scalded in 52 to 55°C water, and the feather were plucked and carcasses sent for regular procedures for viscera removal. 1 male and 1 female chicken whithin the average weight of the each pen (± 10% the standard deviation) were separated and weighed for carcass and cuts yield. Hot carcass weight yield was calculated as a ratio of the live weight of the birds, and breast and thigh meat weight and liver weights were determined and their yields calculated as a ratio of the hot carcass weight. All slaughtered broilers were scored for foot pad dermatitis (**FPD**), according to Goes et al. (2022). Briefly, a scale of 0 to 4 was used, where score 0 represented absence of lesions or dermatitis, 1 very mild evidence, 2 mild evidence of dermatitis, 3 to 4 clear evidence of dermatitis. For data analysis, the average whitin each pen was calculated.

### Sample Collection

Before processing (d 41), on the last day of the experiment and from the same 2 birds, the mid jejunum samples were taken for histological measurements and stored into 10% neutral buffered formalin (Ceylan et al., 2023). Jejunum content and excreta were collected for muramic acid concentration analysis. Cecal content samples were collected and immediately frozen and kept in -20°C for bacteria enumeration.

In addition, 2 to 4 mL of blood was taken from the wing vein, and their serum was separated and frozen at -20°C for liver enzymes analysis. The concentration of enzymes alanine transferase (**ALT**) and alkaline phosphatase (**ALP**) in serum were determined spectrophotometrically by using the enzymes-specific detection kits (Roche Diagnostica, Basel, Switzerland). The absorbance of ALT and ALP were determined at 590 nm wavelength. Concentrations were determined in duplicates of each sample (Tabatabaei et al., 2015). Litter sample was also collected in a sterile 100 mL plastic containers from each floor pens to measure oocyst counts on the last day of the study according to Hauck and Pacheco (2021).

### Muramic Acid Concentration Analysis

Muramic acid (**MurN**) analysis in jejunum content and excreta samples were performed at Novonesis lab by the methodology developed by Novonesis (Frederiksen et al., 2021). Jejunum content samples were analyzed twice, to measure: 1) The amount of soluble muramic acid, and 2) the total amount of muramic acid (the total sample is hydrolysed with acid to make all the muramic acid soluble). Therefore, the % of soluble peptidoglycan (indirectly measured by the amount of muramic acid) was calculated. Muramic acid concentration is a biomarker for peptidoglycan degradation, therefore we can use its quantification (by LC-MS) as an indirect measurement of peptidoglycan break-down.

### Short-Chain Fatty Acids Analysis and Bacteria Count

Short-chain fatty acid (**SCFA**) concentration in the caecum was measured by gas chromatography (Zhang et al., 2003). The cecal bacteria were quantified by using the culture technique (Choi et al., 2009); coliform group in MacConkey agar (105465, Merck, Darmstadt, Germany)), lactic acid bacteria in MRS agar (110660, Merck, Darmstadt, Germany) and total aerobic bacteria counts in Nutrient agar (105450, Merck, Darmstadt, Germany) were determined.

### Statistical Analysis

Statistical analysis of the data obtained in this study was analyzed by ANOVA using the general linear model procedure of MINITAB 18 software (Minitab Ltd.) in a conmpletely randomized block design, with the pen being defined as the experimental unit. The mortality data were subjected to a chi-square test. Probability values *P* ≤ 0.05 were considered significant, and differences among treatments were separated by the Tukey's test.

## RESULTS

### Muramic Acid Concentration

The concentration of soluble and total MurN in the jejunum and excreta are shown in Table 3. The percentage of soluble MurN in the jejunum increased with the supplementation of MUR and Phyto 2 when compared to the other groups (*P* = 0.0001). However, in the excreta, the supplementation of MUR led to the highest concentration of soluble MurN compared to the other groups, which promoted the highest percentage of soluble MurN.

**Table 3:** Effects of muramidase and phytogenics based feed additives supplementation in broiler diets on the level of soluble muramic acid in jejunum and excreta.

Treatments	Jejenum			Excreta		
	Soluble MurN, mg/kg sample	Total MurN, mg/kg sample	% of soluble MurN	Soluble MurN, mg/kg sample	Total MurN, mg/kg sample	% of soluble MurN
Control	119.6	319.5	37.8 b	132.8 b	895.9	14.8 ^c^
MUR	150.6	295.5	54.6 a	194.9 a	793.1	24.6 a
Phyto 1	96.7	317.4	32.3 b	154.3 b	913.2	17.2 ^c^
Phyto 2	146.5	281.8	54.5 a	148.6 b	786.9	22.1 b
*P* value	0.23	0.93	0.0001	0.03	0.43	0.05
Pooled SEM	10.53	23.28	2.19	9.23	34.22	1.55

SEM = Standard Error of Mean (n = 8).

ab Mean with different letters in the same column are significantly different at *P* < 0.05 Tukey's test. MurN: Muramic acid which is a biomarker for peptidoglycan, therefore its quantification (by LC-MS) can be used as an indirect measurement of peptidoglycan.

### Growth Performance, Carcass Yield, and Liver Enzymes

Growth performance results by phase are shown in Table 4. From 0 to 10d and 11 to 23d, it was observed that the supplementation of all feed additives improved the growth performance of the birds. From 23 to 41 d, however, the supplementation of MUR, only, promoted the best FCR when compared to the control group (*P* = 0.04). Considering the entire experimental period (0–41d; Table 5), it was shown that the supplementation of all feed additives improved the BWG (*P* = 0.0001) and the body weight corrected FCR (**BWcFCR**) when compared to the control group. The results of the carcass and cuts yield are shown in Table 6. No differences between the treatments were observed (*P* > 0.05).

**Table 4:** Effects of muramidase and phytogenics based feed additives supplementation in broiler diets on growth performance of broilers in starter, grower, and finisher periods.

Treatment	BW^1^, g	BWG^2^, g	FCR^3^	FI^4^, g	Mortality,%
Starter - 0–10 d					
Control	274.6 b	231.8 b	1.172 a	271.7 b	0.50
MUR	331.9 a	289.0 a	1.150 b	332.5 a	0.88
Phyto 1	332.5 a	289.7 a	1.151 b	333.5 a	0.75
Phyto 2	331.2 a	288.4 a	1.151 b	332.1 a	1.0
*P* value	0.0001	0.0001	0.0001	0.0001	0.80
Pooled SEM	3.00	2.99	0.001	3.22	0.09
Grower - 11–23 d					
Control	929.5 b	654.9 b	1.286 b	841.7 b	0.63
MUR	1033.9 a	702.1 a	1.309 a	919.2 a	0.69
Phyto 1	1029.6 a	697.1 a	1.311 a	913.7 a	0.38
Phyto 2	1025.4 a	694.2 a	1.313 a	911.2 a	0.44
*P* Value	0.0001	0.0001	0.003	0.0001	0.55
Pooled SEM	6.28	3.71	0.003	4.99	0.07
Finisher/Withdrawal - 23 – 41 days					
Control	2563.4 b	1633.9 b	1.812 b	2959.1 b	2.13
MUR	2734.6 a	1700.6 a	1.782 a	3031.1 a	1.88
Phyto 1	2727.1 a	1697.6 a	1.796 ab	3046.9 a	1.63
Phyto 2	2716.4 a	1691.0 a	1.785 ab	3016.5 ab	1.13
*P* value	0.0001	0.0001	0.04	0.006	0.15
Pooled SEM	11.21	8.33	0.005	11.21	0.12

1 Body weight.

2 Body weight gain.

3 Feed conversion ratio.

4 Feed intake. SEM = Standard Error of Mean (n = 8).

ab Mean with different letters in the same column are significantly different at *P* < 0.05 Tukey's test.

**Table 5:** Effects of muramidase and phytogenics based feed additives supplementation in diets of broiler chickens on the overall growth performance (0–41 d).

Treatments	BWG^1^, g	FCR^2^	BWcFCR^3^_2565_	FI^4^, g	Mortality,%	EPEF^5^
Control	2520.6 b	1.620 a	1.629 a	4082.2 b	3.25	367 b
MUR	2691.7 a	1.586 b	1.558 b	4265.9 a	2.87	402 a
Phyto 1	2684.3 a	1.593 ab	1.568 b	4273.9 a	2.75	400 a
Phyto 2	2673.6 a	1.587 b	1.565 b	4243.5 a	2.56	400 a
*P* value	0.0001	0.01	0.0001	0.0001	0.53	0.0001
Pooled SEM	11.16	0.003	0.005	14.96	0.15	2.15

1 Body weight gain.

2 Feed conversion ratio.

3 Body weight corrected FCR.

4 Feed intake.

5 European Poultry Efficacy Factor. SEM = Standard Error of Mean (n = 8).

ab Mean with different letters in the same column are significantly different at *P* < 0.05 Tukey's test.

**Table 6:** Effects of muramidase and phytogenics based feed additives supplementation in broiler diets on carcass and cut yield parameters.^1^

Treatments	Carcass Yield,%	Breast meat,%	Leg Meat,%	Liver,%
Control	71.1	27.6	19.5	2.0
MUR	72.4	28.7	19.1	2.1
Phyto 1	71.0	28.1	18.9	2.0
Phyto 2	70.7	27.2	19.2	2.0
*P* value	0.44	0.13	0.21	0.73
Pooled SEM	0.24	0.17	0.12	0.03

SEM = Standard Error of Mean (n = 8). ^ab^Mean with different letters in the same column are significantly different at P<0.05 Tukey's test.

1 Carcass and cut yield are calculated as ratio of BW.

The relative weight of the liver as well as the concentration of liver enzymes were conducted to determine the hepatoprotection effect of the additives tested. The results of liver weight and enzymes, ALP and ALT analyzed in the blood are shown in Tables 6 and 7, respectively. It was observed that none of these parameters were affected by the dietary treatments (*P* > 0.05).

**Table 7:** Effects of muramidase and phytogenics based feed additives supplementation in broiler diets on the activity of liver enzymes (IU/L).

Treatments	ALP	ALT
Control	286.7	1.505
MUR	341.0	1.855
Phyto 1	301.5	0.832
Phyto 2	395.5	1.312
*P* value	0.97	0.46
Pooled SEM	34.73	0.131

SEM = Standard Error of Mean (n = 8). ^ab^Mean with different letters in the same column are significantly different at *P* < 0.05 Tukey's test. ALP: Alkaline phosphatase; ALT: Alanine aminotransferase.

### Oocyst Counts and Foot Pad Demititis

Since the feed additives used in the present study modify the intestinal microbiota and health, footpad dermatitis score (**FPD**) and oocysts shedding were determined in the trial and the results are shown in Table 8. There was no significant difference between the treatments in terms of excreta oocyst counts (*P* > 0.05). In addition, it was found that the FPD score was significantly decreased by the supplementation of MUR and Phyto 2 to the diets of broiler chickens when compared to control and Phyto 1 groups (*P* = 0.003).

**Table 8:** Effects of muramidase and phytogenics based feed additives supplementation in broiler diets on foot pad dermitites (**FPD**) and Oocyte counts at d 41.

Treatments	FPD	Oocyte counts /g litter
Control	2.57 a	700
MUR	1.86 ^c^	686
Phyto 1	2.07 b	663
Phyto 2	1.87 ^c^	719
*P* value	0.003	0.80
Pooled SEM	0.085	11.32

SEM = Standard Error of Mean (n = 8).

ab Mean with different letters in the same column are significantly different at *P* < 0.05 Tukey's test. FPD: foot pad dermitites.

### Jejunum Histology, Cecal Bacteria Count, and Short-Chain Fatty Acids Concentration

Jejunum morphology, cecal bacteria count and SCFA production results are shown in Tables 9, 10, and 11, respectively. It was observed that the supplementation of MUR increased villus heigh (**VH**) when compared to the control group, or the groups supplemented with phytogenic feed additives (*P* = 0.0001), while maintaing crypt depth (**CD**) similar to the control (*P* = 0.0001); however, the supplementation of phytogenic additives reduced VH and increased CD (Phyto 1 only numerically). These results led to an increased VH/C and villus surface area (**VSA**) increased in the birds supplemented with MUR when compared to the birds supplemented with phytogenics. The number of goblet cells (**GC**) reduced (*P* = 0.002) with the supplementation of Pytho 2 vs. the control group, the supplemeantion of MUR and Pytho 1 resulted in intermediary number of GC.

**Table 9:** Effects of muramidase and phytogenics based feed additives supplementation in broiler diets on jejunum morphology parameters.

Treatments	VH, μm	CD, μm	VW, μm	VH/CD	VSA (mm^2^)	Goblet cell numbers/villus
Control	1364.3 b	142.1 ^c^	190.1 a	9.49 a	0.82 a	222.0 a
MUR	1422.0 a	148.0 ^c^	196.4 a	9.65 a	0.88 a	203.1 ab
Phyto 1	1290.5 ^c^	163.6 b	172.0 b	5.08 b	0.68 b	181.1 ab
Phyto 2	1249.1 ^c^	178.8 a	162.1 b	5.59 b	0.63 b	163.8 b
*P* value	0.0001	0.0001	0.03	0.01	0.0001	0.002
Pooled SEM	11.75	3.22	3.86	0.44	0.020	5.28

SEM = Standard Error of Mean (n = 8).

ab Mean with different letters in the same column are significantly different at *P* < 0.05 Tukey's test. VH: Villus height, CD: Crypt depth, VW:Villus width, VSA:Villus surface area.

**Table 10:** Effects of muramidase and phytogenics based feed additives supplementation in broiler diets on cecal bacteria counts.

Treatments	Total aerobe counts (*log cfu/g*)	*Lactobacillus* (*log cfu/g*)	Coliforms (*log cfu/g*)
Control	7.88 b	7.66 b	7.55
MUR	9.49 a	9.53 a	7.20
Phyto 1	8.03 b	7.51 b	7.50
Phyto 2	8.18 ab	7.74 b	7.60
*P* value	0.009	0.0001	0.85
Pooled SEM	0.19	0.18	0.18

SEM = Standard Error of Mean (n = 8).

ab Mean with different letters in the same column are significantly different at *P* < 0.05 Tukey's test.

**Table 11:** Effects of muramidase and phytogenics based feed additives supplementation in broiler diets on cecum short chain fatty acids (**SCFA**) concentration (mmol/L).

Control	Acetate	Propionate	Butyrate	Isobutyrate	Valerate	Isovalerate	BCFA	Total SCFA
Control	27.2 b	8.2 b	7.2 b	0.85 b	0.96	0.88 b	2.69 ^c^	45.3 ^c^
MUR	35.6 a	11.9 a	10.3 a	1.24 a	1.29	1.18 a	3.71 a	61.5 a
Phyto 1	30.5 b	9.4 b	8.9 ab	0.88 b	1.12	0.99 ab	2.99 ^bc^	51.7 b
Phyto 2	27.4 b	9.6 b	8.2 b	1.03 ab	1.16	1.02 ab	3.22 b	48.5 ^bc^
*P* value	0.0001	0.0001	0.001	0.005	0.06	0.002	0.000	0.0001
Pooled SEM	0.67	0.29	0.285	0.04	0.04	0.03	0.073	1.05

SEM = Standard Error of Mean (n = 8).

ab Mean with different letters in the same column are significantly different at *P* < 0.05 Tukey's test. BCFA: Branch chain fatty acids (iso-butyric12-me-butyric1iso-valeric).

Additionally, the supplementation of MUR promoted the proliferation of the total aerobe bacteria (*P* = 0.009) and *Lactobacillus* (P = 0.0001), when compared to the other groups. Lastly, the supplementation of MUR increased the cecal concentration of acetate, propionate, butyrate, isobutyrate, isovalerate, total BCFA, and total SCFA when compared to the control group or the groups supplemented with phytogenic feed additives (*P* < 0.05).

## DISCUSSION

Intestinal health feed additives are routinely used in poultry feeds aiming to support the growth performance, intestinal health, welfare and well-being of the animals. It has become clear over the years, following the increased restrictions in the use of AGPs, that the non-AGP alternatives have varied mechanisms of actions and that their success will depend on specific conditions within each situation. In this regard, it is essential for nutrition and health companies to deploy resources to develop innovative and precise solutions to tackle the variaty of challenges that broiler producers constantly face. The deeper understanding of how different solutions work in the animals, especially under commercial-like conditions, is of paramount importance in filling gaps of knowledge not studied before. In this sense, we investigated the differences between feed additives (MUR and phytogenics) with clear variation in their mechanisms of action on the growth performance and gastrointestinal functionality of broiler chickens, and observed that both MUR and phytogenic feed additives led to improved growth performance of broiler chickens. However, MUR, only, increased the percentage of soluble MurN in both jejunum content and excreta, which is a property intrinsically associated with the mechanism of action of MUR, wherein by degrading PGNs, and generating muropeptides, it elicits an anti-inflammatory response in chickens (Traub et al., 2006; Wang et al. 2021; Bortoluzzi et al., 2024). In a similar manner, the phytogenic 2 product, composed of alkaloids, only increased the percentage of soluble of MurN in the jejunal content, most likely due to the antimicrobial activity of such compounds (Kikusato, 2021), and therefore, the release of PGN from the bacterial cell wall.

The enhancement in the growth performance of broiler chickes supplemented with MUR and phytogenic feed additives and under different challenge conditions has been documented (Kikusato, 2021; Brugaletta et al., 2022; Goes et al., 2022; Khongthong et al., 2023; Bortoluzzi et al., 2023, 2024; Ceylan et al., 2023; Cho et al., 2024). The increase in the abudance of *Lactobacillus* in the cecal content by the supplementation of MUR had a positive effect of the growth performance of the animals and is in agreement with previous publications ((Lichtenberg et al., 2017; Bortoluzzi et al., 2023). Additionally, Brugaletta et al. (2022) observed that, besides improving the growth performance, the supplementation of MUR led of several changes in the metabolic profile of the cecal content and blood of chickens. In a similar direction, we presented herein the capacity of the MUR enzyme in affecting the production of SCFA. We observed that the supplementation of MUR increased the production of most of SCFA evaluated, showing that by the immunomodulation effect of MUR, a beneficial selected microbiome leads to increased production of SCFA. For instance, we reported that propionate and butyrate were increased by over 30% with MUR supplemented. Propionate is quickly absorbed by the epithelial cells and taken up by the liver where is serves as a substrate for gluconegenesis (Schwiertz et al., 2010). Butyrate, on the other hand, is an important source of energy for intestinal epithelial cells and has several effects on the host, including, but not limited to regulation in the production of cytokines, antimicrobial peptides, and mucins, profileration, maturation and differentiation of epithelial cells, among others (Guilloteau et al., 2010; Bortoluzzi et al., 2017, 2022).

Phytogenic feed additves have been widely studied as AGPs alternatives. Studies have demonstrated beneficial effects of the bio-active plant compounds in broiler chickens (Lee et al., 2011; Almeida et al., 2012; Kim et al., 2013; Kikusato, 2021; Khongthong et al., 2023). Although the exact mechanism of action of phytogenics is highly different depending on the active compound, it is believed to include disruption of the cellular membrane of pathogens, modification of cells affecting the virulence capacity of the microorganism, stimulation of the immune system, and protection against pathogen binding to the intestinal mucosa (Diaz-Sanchez et al., 2015). Among the phytogenics, essential oils (EO) have been subject of many studies mainly due to their antimicrobial and growth promoter effects. The mechanism of action of EO is based on its chemical composition, and on the location of the hydroxyl group; for example, thymol and carvacrol are isomeric molecules and possess similar antimicrobial effects (Diaz-Sanchez et al., 2015). Despite the broad range of beneficial effects on the host animals, including the enhancement of the growth performance, in the present study, both phytogenic groups had a distinct effect on the intestinal morphology when compared to MUR. It was observed a reduction of villus height and width, increase in crypt depth, and consequently reduction in villus to crypt ration and the villus surface area. The effects may reflect the beneficial anti-inflammatory and immunomodulatory effects of phytogenics on the host, including induction of heat shock proteins, induction of Toll-like receptors, and induction of proliferation and maturation of T-Helper cell (Th-1 and Th-2) to maintain a balance between cellular and humoral immune response (Hashemi and Davoodi, 2012).

In this context and by specifically degrading PGNs, the anti-inflammatory role exerted by MUR on the GIT of chickens (Bortoluzzi et al., 2024), has been translated into enhanced digestion and absorption capacity in chickens with improved nutrient digestibility (Goodarzi Boroojeni et al., 2019; Sais et al., 2020; Goes et al., 2022; Pérez-Calvo et al., 2023). In the present experiment, nutrient digestion and absorption were not evaluated, but chickens supplemented with MUR showed longer VH compared to control which could favor nutrient absorption. Additionally, a less viscous intestinal content as well as a more beneficial microbiota have been reported with the inclusion of MUR in the diets of broiler (Bortoluzzi et al., 2023). Nevertheless, the improved overall gastrointestinal health of chickens supplemented with MUR and phytogenic 2, to some extent, may have contributed to better litter quality leading to lower incidence of lesions characteristic of FPD which is a deformation of the foot keratin tissue formed due to sticky droppings, wet litter, and the presence of uric acid in the litter. This measure is a direct indicator of the health of the digestive system of the chickens, as also highligthed by previous publications (Pirgozliev et al., 2021; Brugaletta et al., 2022; Goes et al., 2022).

While the broad action of phytogenics makes them important strategies when looking for AGP replacements, unless otherwise defined, they may be less suitable when a specific issue needs to be targeted with a precise solution, which is the case of MUR. Therefore, with a practical approach, we concluded that the supplementation of MUR and phytogenics to the diets of broiler chickens improved the growth performance. Muramidase, only, was capable of effectively degrading PGNs in jejunum and excreta, as well as to imporve intestinal morphology, increase the abundance of *Lactobacillus* and SCFA production. The targeted benefits of MUR makes it an impactful strategy in the search for more efficient and sustainable dietary solutions to the poultry industry.

## DISCLOSURES

CB, EPC, OG are employed by dsm-firmenich. The other authors declare no conflict of interest.
